# The Impact of Weather and Seasons on Falls and Physical Activity among Older Adults with Glaucoma: A Longitudinal Prospective Cohort Study

**DOI:** 10.3390/s21103415

**Published:** 2021-05-14

**Authors:** Hursuong Vongsachang, Aleksandra Mihailovic, Jian-Yu E, David S. Friedman, Sheila K. West, Laura N. Gitlin, Pradeep Y. Ramulu

**Affiliations:** 1 Wilmer Eye Institute, Johns Hopkins University School of Medicine, Baltimore, MD 21287, USA; hvongsa2@jhmi.edu (H.V.); amihail2@jhu.edu (A.M.); je1@jhu.edu (J.-Y.E.); shwest@jhmi.edu (S.K.W.); 2 Department of Epidemiology, Johns Hopkins Bloomberg School of Public Health, Baltimore, MD 21205, USA; 3 Massachusetts Eye and Ear, Harvard Medical School, Boston, MA 02114, USA; david_friedman@meei.harvard.edu; 4 College of Nursing and Health Professions, Drexel University, Philadelphia, PA 19102, USA; lng45@drexel.edu

**Keywords:** falls, accelerometer, older adults, seasons, weather

## Abstract

Understanding periods of the year associated with higher risk for falling and less physical activity may guide fall prevention and activity promotion for older adults. We examined the relationship between weather and seasons on falls and physical activity in a three-year cohort of older adults with glaucoma. Participants recorded falls information via monthly calendars and participated in four one-week accelerometer trials (baseline and per study year). Across 240 participants, there were 406 falls recorded over 7569 person-months, of which 163 were injurious (40%). In separate multivariable regression models incorporating generalized estimating equations, temperature, precipitation, and seasons were not significantly associated with the odds of falling, average daily steps, or average daily active minutes. However, every 10 °C increase in average daily temperature was associated with 24% higher odds of a fall being injurious, as opposed to non-injurious (*p* = 0.04). The odds of an injurious fall occurring outdoors, as opposed to indoors, were greater with higher average temperatures (OR per 10 °C = 1.46, *p* = 0.03) and with the summer season (OR = 2.69 vs. winter, *p* = 0.03). Falls and physical activity should be understood as year-round issues for older adults, although the likelihood of injury and the location of fall-related injuries may change with warmer season and temperatures.

## 1. Introduction

Falls are the leading causes of non-fatal and fatal injuries in the older population [1,2]. More than one in four adults above 65 years of age experience a fall annually [3]. Falls can lead to various negative physical and mental consequences for the individual, including fractures, disability, restriction of activity, and fear of falling [4,5], as well as enormous healthcare costs for society [6]. In addition to falls, another important issue for the overall health, disease prevention, quality of life, and healthcare burden for older adults is physical activity [7]. Falls can negatively impact physical activity [8]. Maintaining physical activity is particularly important in older adults, as physical activity declines with age and many older adults do not meet recommended physical activity levels [9].

In climates with varying weather across seasons, it is important to understand how fall rates and physical activity change across seasons both clinically and in research studies. Specifically, understanding periods of the year associated with a higher risk for falling and lower levels of physical activity could guide individuals on how to preserve physical activity and prevent falls throughout the year. Prior work has suggested a relationship between weather and seasons with falls, fall-related injuries [10,11,12,13,14,15,16,17], and physical activity [18,19,20,21,22,23]. However, literature describing how falls and physical activity vary across weather conditions and seasons is still limited. Notably, the relationships between weather conditions, seasons, and either falls or physical activity remain understudied in high-risk populations, such as those with visual impairment, who may be at greater risk for falls [24,25,26] and decreased physical activity [27,28,29,30,31,32] as compared to their normally sighted peers.

Here, we aim to examine the seasonality and weather variability of falls and physical activity with a longitudinal prospective cohort of older adults with visual field (VF) loss from glaucoma. Specifically, we aim to answer whether: (1) falls and fall-related injuries are associated with weather and season, (2) whether physical activity, measured by daily steps taken and active minutes, vary with weather and seasons, and (3) whether VF loss modifies the relationship between weather and seasons on falls and physical activity. Our study was conducted in the Mid-Atlantic United States, where the average high temperature varies by 45 °F (25 °C) over the course of the year [33]. We hypothesized that there would be no associations between weather and seasons with either falls or physical activity.

## 2. Materials and Methods

The “Falls in Glaucoma” study protocol received approval by the Johns Hopkins Medicine Institutional Review Board. Methods for this prospective cohort have been previously described in detail [34,35,36,37].

### 2.1. Study Participants

Participants were recruited from the Johns Hopkins Wilmer Eye Institute glaucoma clinic between 2013–2015 for a three-year study. Eligible patients were at least 57 years of age (would be 60 years old by the end of the three-year study period), could perform vision assessments, lived within 60 miles of the hospital, and had a clinical diagnosis of glaucoma or glaucoma suspect. Exclusion criteria included: (1) neovascular or uveitic glaucoma, (2) visual acuity worse than 20/40 in either eye due to conditions other than glaucoma, (3) hospitalization within the last month, and (4) surgery within the last two months. All study participants gave written informed consent.

### 2.2. Falls and Follow-Up Data Collection

Participants recorded daily information on fall occurrences using a falls calendar, which was turned into the study team monthly over the three-year study period. Falls were defined for participants as unintentionally landing on the ground or a lower level and illustrated using an instructional video provided at the baseline visit. The study team contacted participants weekly to retrieve missing calendars. Calendars that were not received within 3 months were recorded as missing data. For each participant, up to 36 months of falls data were included in this study.

Within two weeks of receiving the calendars, the study team administered a 30-item, follow-up, phone questionnaire to participants who indicated experiencing a fall to record location (i.e., indoors vs. outdoors), context, injury, treatment sought, and brief narratives about each fall. Falls associated with pain, bruising, swelling, pulled muscle, sprained ligaments/tendons, joint dislocation, or broken bone/fracture were classified as injurious.

### 2.3. Physical Activity Data Collection

Minute-to-minute steps and activity information were obtained from an omni-directional accelerometer (Actical, Respironics Inc., Murrysville, PA, USA) that participants wore clipped to their waist for one week following the baseline visit and once every study year thereafter. A study team member provided at least two reminder calls to participants during that week to reinforce accelerometer wear adherence. Accelerometer data were used from participants who wore the device for a minimum of 8 h/day for at least 4 days during each trial. For each person, total daily steps and non-sedentary active minutes (the sum of light, moderate, and vigorous activity) were averaged over valid days for each accelerometer trial.

### 2.4. Weather and Seasons

Daily summaries of weather information, including average temperature and total precipitation (mm), from the Baltimore Washington International Airport weather station (Station USW00093721, 39.1733°, −76.684°) between 2013 and 2018 were obtained from the National Centers for Environmental Information [38] and merged with participant data for corresponding dates. Seasons were assigned to each study day based on Northern Hemisphere astronomical dates (e.g., dates between 22 September and 21 December 2013 constituted Autumn, 22 December 2013 to 20 March 2014 constituted Winter, 21 March to 21 June 2014 constituted Spring, and 22 June to 22 September 2014 constituted Summer). For each person, the average daily temperature and precipitation across all days in each accelerometer trial was calculated.

### 2.5. Visual Field Data Collection

All participants received baseline visual assessments, including visual field testing, visual acuity (VA), and contrast sensitivity tests. After obtaining right and left eye visual fields on a Humphrey 24-2 perimeter (Carl Zeiss Meditec, Dublin, CA, USA), integrated VF (IVF) was calculated by taking the maximum sensitivity in decibels at corresponding coordinates across the right and left eye 24-2 VF tests, averaging the raw values of these sensitivities, and then converting back to decibels. Participants were categorized as having mild (>28 dB), moderate (23–28 dB), or severe (<23 dB) VF damage based on IVF sensitivity [39]. Letters read on a back-lit ETDRS chart at 4 m and a MARS chart at 40 cm (Mars Perceptrix, Chappaqua, NY, USA) were used to derive visual acuity (VA) and contrast sensitivity (CS), respectively. Testing was done with patients wearing their usual corrective lenses and results were converted into log units as logMAR (log of the minimum angle of resolution) and logCS, respectively.

### 2.6. Covariates

Participants completed standardized questionnaires that included age, gender, race, and number of non-visual comorbidities related to falling. Polypharmacy, the use of 5 or more systemic prescription medications, was recorded by directly observing each participant’s pill bottles or by participant report. Grip strength was tested by asking participants to use their dominant hand to squeeze the Jamar Hand Dynamometer (Sammons Preston Rolyan, Bolingbrook, IL, USA) 3 times. To test leg strength, patients were instructed to resist the pressure of the MircoFET2 Dynamometer (Hoggan Scientific LLC, West Jordan, UT, USA) placed above their knee while sitting and not allowing their foot to touch the ground. Strength testing was done twice for a 5-s period. Grip and leg strength were taken from the maximum values of each test in kilograms of force.

### 2.7. Statistical Analysis 

Participant demographics and baseline health and vision characteristics were tabulated as frequencies and percentages, means and standard deviations (SD), or medians and interquartile ranges (IQR), as appropriate.

Temperature, precipitation, and season were included as exposure variables in separate models analyzing the association between each exposure variable with falls, injurious falls, daily steps, and daily activity minutes. All models analyzing fall-related outcomes utilized multivariable logistic regression models incorporating general estimating equations (GEE) to account for clustering across study days within the same individual. Separate models were constructed to examine the association between each weather or season variable with (1) the odds of falling for each person-day, (2) the odds of a recorded fall occurring outdoors vs. indoors, (3) the odds of an observed fall being injurious vs. non-injurious, and (4) the odds of a recorded injurious fall occurring outdoors vs. indoors. Average daily steps and active minutes for each yearly trial by person were also modeled against the average temperature, precipitation, or season over the trial week using multivariable negative binomial regressions employing GEE to account for correlations across study years for the same person, with outcomes expressed as rate ratios (RR). All models were controlled for age, race, sex, IVF sensitivity, number of comorbidities, and polypharmacy, based on prior research demonstrating the association of these covariates with falls, balance, and gait [34,36]. Analyses were performed in Stata/SE 15.1 (StataCorp, College Station, TX, USA).

## 3. Results

### 3.1. Study Cohort

Two-hundred forty-five participants were enrolled in the study. Participants were excluded from falls analysis based on neurological conditions related to falls (*n* = 2), insufficient fall data (*n* = 2), and missing VF data (*n* = 1), leaving 240 participants for inclusion. For physical activity analysis, participants were excluded for missing VF data (*n* = 1) and insufficient accelerometer data (*n* = 1), which left 243 participants for accelerometer analysis. Across 244 participants, excluding one individual who was missing VF data in both falls and physical activity analysis, the mean age upon entry into the study was 70.6 years (SD = 7.6), 51% were male (*n* = 125), and 29% were African-American (*n* = 70) (Table 1). Median IVF sensitivity was 28 dB [IQR: 26.0–29.7 dB] (normal vision is ≥31 dB), while mean better and worse eye deviations were −2.5 [IQR = −5.4 to −0.7 dB] and −5.7 [IQR = −12.9 to −2.8], respectively. One hundred twenty-one (49.6%) participants had mild, 98 (40.2%) participants had moderate, and 25 (10.3%) participants had severe VF damage.

### 3.2. Falls 

**Figure 1:**
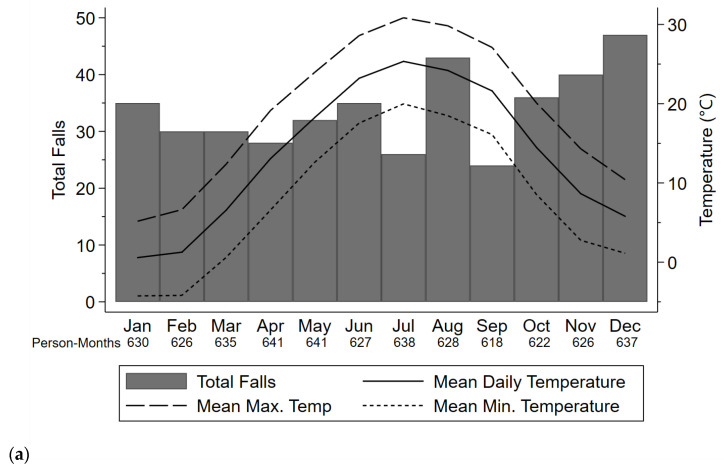

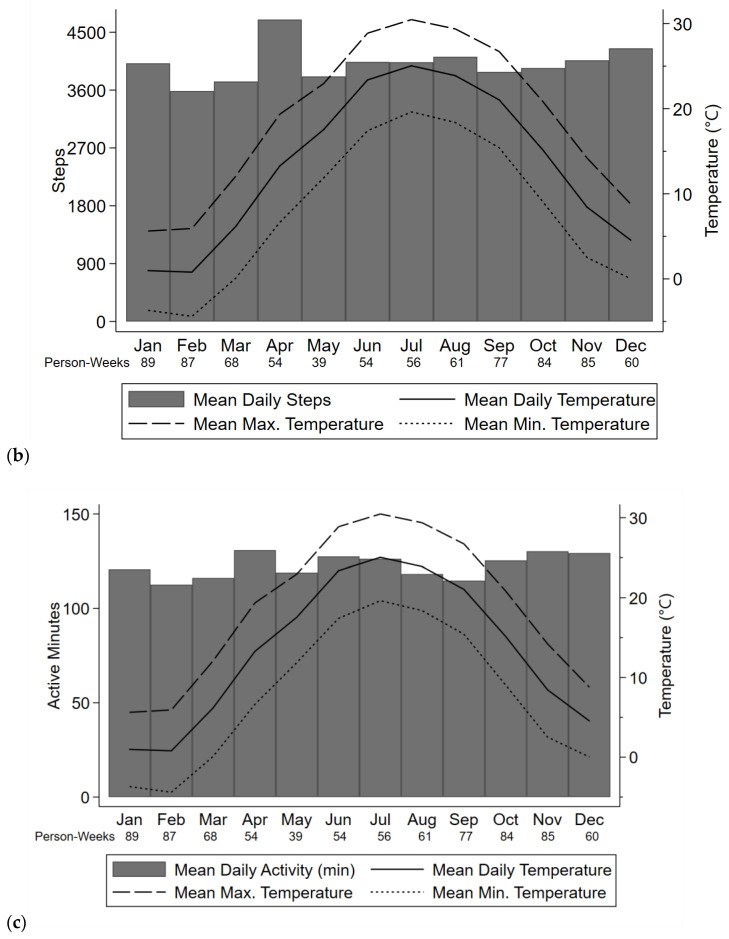
(**a**) Total aggregate falls, (**b**) mean daily steps, and (**c**) mean daily active minutes across all study years and all participants. Daily average, maximum, and minimum temperatures displayed were averaged across all study years.

**Figure 2:**
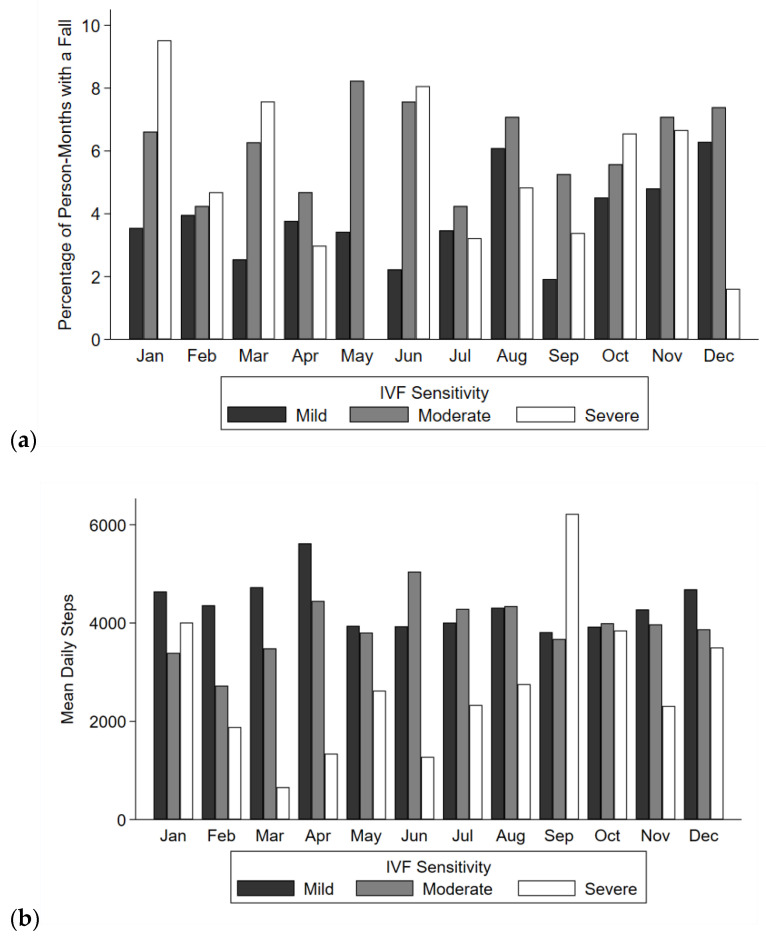

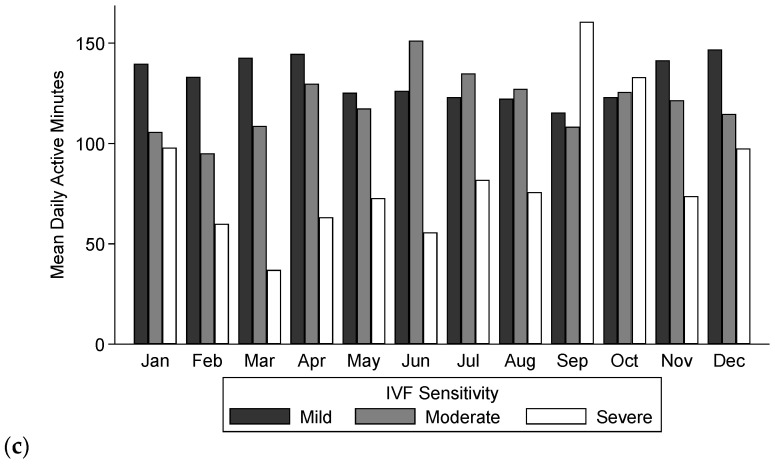
(**a**) Percent of months with a fall, (**b**) mean daily steps, and (**c**) mean daily active minutes by integrated visual field (IVF) severity group and month across all participants and the full study period. VF damage was categorized as mild (>28 dB in the integrated visual field), moderate (23–28 dB), or severe (<23 dB). (**a**) Approximately 114–118, 96–97, and 24–25 persons per month contributed to mild, moderate, and severe groups, respectively. (**b**,**c**) Per month, there were 19–53 person-weeks in the mild group, 17–41 person-weeks in the moderate group, and 2–14 person-weeks in the severe group.

A total of 406 falls were recorded (Figure 1a) over 7569 person-months across 240 participants. In total, 91 (22%) falls occurred in the Spring, 102 (25%) falls occurred in the Summer, 113 (28%) falls occurred in the Autumn, and 100 (25%) falls occurred in the Winter. Across all study days in the falls data, 26% of study days (*n* = 58,814) belonged to the Spring, 26% belonged to the Summer (*n* = 58,673), 24% belonged to the Autumn (*n* = 55,516), and 24% belonged to the Winter (*n* = 56,055). Of the 380 (93.5%) falls with location information recorded, 195 falls occurred indoors (51%) and 185 (49%) occurred outdoors. In separate multivariable GEE models, there was no significant association between temperature, precipitation, or season with the odds of falling (*p* > 0.09 for all) (Table 2). Additionally, the likelihood of a fall occurring outdoors vs. indoors did not differ with temperature, precipitation, or seasons (*p* > 0.07 for all) (Table 2). No significant interactions were observed between IVF sensitivity and temperature, precipitation, or season as related to falls (*p* > 0.08 for all) (Figure 2a).

### 3.3. Injurious Falls 

**Figure 3:**
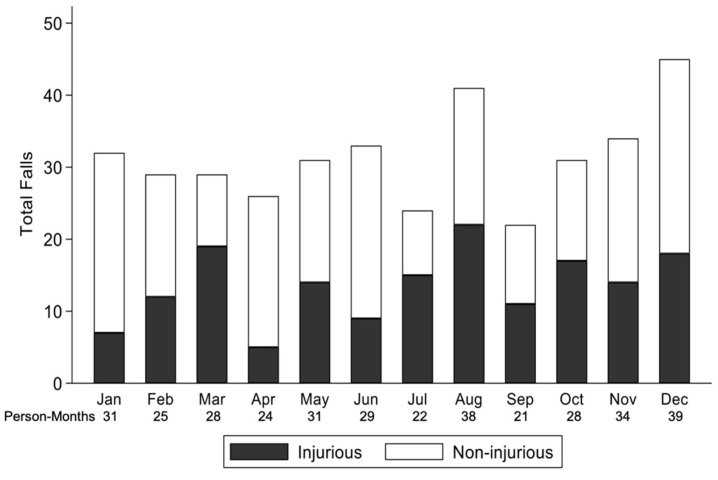
Total falls, classified as injurious vs. non-injurious falls, by month across all study years and study participants.

Out of 406 falls, 377 falls (93%) had follow-up information recorded about the context of the fall, of which 43% (*n* = 163) resulted in injuries (Figure 3). Eighty-four (51.5%) of these falls occurred outdoors. Among the 163 injurious falls, 27 (17%) occurred in the Spring, 50 (31%) occurred in the Summer, 45 (28%) occurred in the Autumn, and 41 (25%) occurred in the Winter. There were 14 (52%) fall-related injuries that occurred outdoors in the Spring, 33 (66%) that occurred outdoors in the Summer, 20 (44%) that occurred outdoors in the Autumn, and 17 (41%) that occurred outdoors in the Winter. In adjusted GEE models, every 10 °C increase in average daily temperature was associated with 24% higher odds of a fall being injurious, as opposed to non-injurious (OR = 1.24, 95% confidence interval (CI) [1.01, 1.53], *p* = 0.04) (Table 2). The odds of an injurious fall occurring outdoors, as opposed to indoors, were greater on days with higher average temperatures (OR = 1.46 per 10 °C, 95% CI [1.03, 2.07], *p* = 0.03) and during the summer months (OR = 2.69 in Summer vs. Winter, 95% CI [1.12, 6.50], *p* = 0.03). There were no significant interactions between IVF sensitivity and temperature, precipitation, or season in association with injury occurring in the context of a fall (*p* > 0.36 for all).

### 3.4. Daily Steps and Active Minutes 

There were a total of 814 person-weeks recorded with accelerometer data across 243 participants. A total of 222 weeks (27%) were recorded in the Winter, 152 weeks (19%) were recorded in the Spring, 196 weeks (24%) were recorded in the Summer, and 244 weeks (30%) were recorded in the Autumn. Taking the mean across each participant’s daily averages for all trials across all years, the cohort averaged 3994 steps/day (SD = 2649 steps/day) and 122 active minutes/day (SD = 63 active minutes/day) (Figure 1b,c). In adjusted negative binomial GEE models, average daily steps and average daily activity did not significantly vary with temperature, precipitation, or seasons (*p* > 0.35 for all) (Table 3). IVF sensitivity did not show significant interactions with temperature, precipitation, or season with respect to steps and activity (*p* > 0.23 for all) (Figure 2b,c).

## 4. Discussion

In this prospective cohort of older adults with VF damage followed over three years, neither fall risk nor physical activity varied with weather or season. However, there was some evidence that falls were more likely to result in injury with warmer temperatures. Injurious falls were also more likely to occur outdoors, as opposed to indoors, with warmer temperatures and seasons as well.

The relationship between falls and weather or seasons has been studied previously, with varying conclusions. While many studies have reported an association between falls and colder, winter weather conditions among older people [10,11], some studies have found no patterns in the temperature or seasonality of falls among older individuals, including studies conducted in northern regions [40,41] and a study using national emergency department surveillance data in the U.S. [17]. Differences in the measurement of exposures and outcomes may contribute to the different conclusions found between our study and prior work that have reported a meteorological or seasonal pattern to falls. For example, other studies have used recall of past falls or chart review to collect data on fall occurrences [10,11]. Some also grouped cold and warm months generally, used meteorological seasons, or did not include specific factors, such as temperature or precipitation on a day-to-day basis [10,11].

A possible explanation behind the lack of temperature and season variability with fall occurrence may be the relative lack of exposure to weather or seasonal changes experienced by older individuals. Older adults spend the majority of time indoors at home [42]. Along those lines, prior work in this cohort showed that most falls occurred around the home, either inside or immediately outside [37]. Vikman et al. used a similar methodology to the present work where they utilized a monthly falls calendar for one year among older adults receiving home help services, and did not find a relationship between falls and temperature [41]. Combined together, this suggests that older adults may be relatively insulated from the effects of weather and seasons if they are spending the majority of time at home regardless of the time of year.

While falls were generally not associated with weather or seasons, our results suggest that warmer weather is associated with an increased likelihood of a fall being injurious. Additionally, an injurious fall was more likely to occur outdoors, as opposed to indoors, in warmer conditions. This is consistent with prior work in this cohort that found that falls occurring outdoors were more likely to result in injury, as opposed to indoor falls, and that significant contributors were wet ground and uneven surfaces [37]. The association between warmer weather and injurious falls could be related to warmer weather or summer seasons encouraging or permitting outdoor activities with more dangerous conditions conducive for injuries. For example, the third and fourth most common locations of outdoor falls previously identified in this cohort were the yard/garden and park/forest/beach [37]. Educating older individuals on safety precautions during outdoor activities, while balancing the benefits of spending time outdoors, may be helpful to prevent injurious falls [43].

Notably, our finding of the positive association between warmer weather and injurious falls contrasts with other work reporting a higher risk of fall-related injuries in winter or colder seasons in both northern and southern hemispheres, possibly attributed to slipping on ice or snow [12,13,15,16]. One key difference is that these studies defined injury as a fracture, of which cases were often detected through medical records, while the present study emphasized all injuries, including bruising or pain, as reported via a monthly calendar. Another study including injuries ranging from superficial (e.g., bruises, lacerations) to more severe injuries (e.g., fractures, intracranial head injuries) also found no seasonal variation with fall-related injuries, although temperature was not included as a variable in that analysis [40]. Differences in study design, such as reporting of population-based incidence rates, categorization of cold versus warm months, and study climate may also contribute to the different conclusions between prior work and this study on the weather and seasonal patterns of fall-related injuries. More work is needed to determine the relationship between weather and seasons and likelihood of injuries related to falls.

As for physical activity, we did not find an association between weather or season with steps and active minutes. Prior research investigating the association between weather and seasons with physical activity, as measured by accelerometer or pedometer trials among community-dwelling older adults, suggest decreased walking with snow and precipitation and greater physical activity with increased temperatures and warmer months [18,19,20,21,22,23]. Our finding of no association could once again be related to the indoor habits of older people in this cohort/geographic region, limiting the impact of these exposures on activity. Light activity, which constituted the majority of non-sedentary active minutes used in our study, may also not be impacted much by weather or seasons. Additionally, the cohort may be relatively less active overall as compared to normally sighted individuals [29], although many of our participants had normal vision or mild VF damage. Of note, the lack of association between physical activity and weather or seasons was consistent across both recorded steps and active minutes, with multiple trials per person, whereas prior work mostly reported on either steps or active minutes with more limited data.

Finally, we found no interaction between IVF sensitivity and weather and seasons in relation to falls and physical activity. This suggests that weather and seasons do not impact those with greater VF loss more as compared to those with no or mild VF loss. As such, these results may apply more broadly to all older adults.

Some limitations should be noted. There could be selection bias from those who are more prone to falling participating in the cohort. These results may not be generalizable to all climates. Furthermore, with Baltimore’s mild temperate climate [33], there may be relatively fewer climate-related concerns or extreme variations in weather conditions and seasons in comparison to other climates, limiting the detected impact of these exposures on falls and physical activity. Future studies with a greater number of falls and active minutes may also be better able to characterize the effect of season and weather on these outcomes. Additionally, our analysis did not include other factors, such as neighborhood environment, that may be relevant for the relationships between season and weather with falls or physical activity. Nonetheless, our study was able to analyze falls recorded with the gold standard method [44] and objectively measured physical activity against weather and seasons on a day-to-day basis in a prospective cohort. Finally, we did not develop a risk calculator for falls or physical activity incorporating weather and patient variables as there was a lack of positive associations to model in our study. However, a risk calculator in the form of a digital health application for older adults may be an area of investigation for future work.

## 5. Conclusions

In conclusion, this study found that, generally, weather and seasons may not be important determinants of falls and physical activity in older adults. However, older adults may need to be cognizant of fall-related injury prevention techniques during warmer weather, particularly when outdoors. Overall, while providers are still encouraged to counsel on the dangers of climate-related conditions like ice or snow, these findings suggest that fall prevention and physical activity promotion should be treated as year-round issues for older adults.

## Figures and Tables

**Table 1:** Study population characteristics at baseline.

Characteristics	Values (*n* = 244)
Age (years), mean (SD)	70.6 (7.6)
Male, *n* (%)	125 (51.2)
African-American race, *n* (%)	70 (28.7)
Living alone, *n* (%)	49 (20.1)
Employed, *n* (%)	87 (35.7)
Education *, *n* (%)	
Less than high school	8 (3.3)
High school	29 (11.9)
Some college	33 (13.5)
Bachelor’s degree	59 (24.2)
Higher than Bachelor’s degree	114 (46.7)
Health	
Comorbid illnesses >1, *n* (%)	159 (65)
Polypharmacy, *n* (%)	81 (33)
Body mass index (kg/m^2^), mean (SD)	27.2 (5.1)
Grip strength (kg), mean (SD)	31.6 (10.3)
Lower body strength (kg), mean (SD)	17.7 (6.0)
Vision, median (IQR)	
IVF sensitivity (dB)	28 (26.0, 29.7)
Mean deviation better eye	−2.5 (−5.4, −0.7)
Mean deviation worse eye	−5.7 (−12.9, −2.8)
Better eye visual acuity-logMAR	0.1 (0, 0.2)
Binocular log CS	1.7 (1.6, 1.8)

SD = standard deviation, *n* = number, kg = kilogram, m = meter, IVF = integrated visual field, dB = decibel, IQR = interquartile range, logMAR = logarithm of the minimum angle of resolution, CS = contrast sensitivity. * One participant is missing education data.

**Table 2:** Association of weather and season variables with likelihood and location of falls and injurious falls (N = 240) ^1^.

	Likelihood of Falling		Likelihood a Fall Is Injurious vs. Non-Injurious		Likelihood of Fall Occurring Outdoors vs. Indoors		Likelihood of Injurious Fall Occurring Outdoors vs. Indoors	
Weather Variable	OR (95% CI)	*p*	OR (95% CI)	*p*	OR (95% CI)	*p*	OR (95% CI)	*p*
Temperature								
Average daily temperature per 10 °C increment	0.92 (0.84, 1.02)	0.12	1.24 (1.01, 1.53)	0.04	1.03 (0.84, 1.26)	0.76	1.46 (1.03, 2.07)	0.03
Average daily temperature > 10 °C vs. ≤10 °C	0.84 (0.69, 1.02)	0.09	1.42 (0.92, 2.18)	0.11	1.02 (0.67, 1.54)	0.94	1.91 (0.98, 3.69)	0.06
Precipitation								
Daily precipitation (mm)	1.05 (0.82, 1.35)	0.70	1.28 (0.68, 2.41)	0.44	0.56 (0.29, 1.09)	0.09	0.48 (0.17, 1.38)	0.18
Presence of precipitation vs. no precipitation	1.12 (0.92, 1.37)	0.27	0.96 (0.62, 1.47)	0.84	0.74 (0.48, 1.13)	0.16	1.24 (0.64, 2.40)	0.52
Season								
Spring vs. Winter	0.87 (0.65, 1.15)	0.33	0.61 (0.33, 1.13)	0.12	0.57 (0.31, 1.04)	0.07	1.53 (0.56, 4.16)	0.41
Summer vs. Winter	0.98 (0.74, 1.29)	0.90	1.55 (0.86, 2.77)	0.14	1.15 (0.64, 2.06)	0.65	2.69 (1.12, 6.50)	0.03
Autumn vs. Winter	1.15 (0.88, 1.51)	0.31	1.11 (0.62, 1.98)	0.73	0.79 (0.44, 1.41)	0.43	1.11 (0.46, 2.66)	0.82

^1^ Controlled for age, race, sex, IVF sensitivity, number of comorbidities, and presence of polypharmacy. OR = odds ratio.

**Table 3:** Association of weather and season variables with average daily steps and non-sedentary active minutes (N = 243) ^1^.

	Average Daily Steps		Average Daily Active Minutes	
Weather Variable	RR (95% CI)	*p*	RR (95% CI)	*p*
Temperature				
Average daily temperature per 10 °C increment	1.01 (0.94, 1.09)	0.77	1.01 (0.94, 1.09)	0.77
Average daily temperature > 10 °C vs. ≤10 °C	1.00 (0.87, 1.16)	0.95	1.01 (0.88, 1.17)	0.88
Precipitation				
Average daily precipitation (mm)	1.11 (0.72, 1.71)	0.63	0.93 (0.59, 1.44)	0.73
Presence of precipitation vs. no precipitation	0.97 (0.80, 1.18)	0.77	0.94 (0.77, 1.15)	0.57
Season				
Spring vs. Winter	1.10 (0.90, 1.34)	0.35	1.07 (0.87, 1.31)	0.54
Summer vs. Winter	1.03 (0.85, 1.26)	0.74	1.04 (0.85, 1.28)	0.69
Autumn vs. Winter	1.07 (0.90, 1.28)	0.44	1.07 (0.90, 1.29)	0.44

^1^ Controlled for age, race, sex, IVF sensitivity, number of comorbidities, and presence of poly-pharmacy. RR = rate ratio.

## Data Availability

Data can be made available upon reasonable request.
